# The Prevalence and Predictors of Sleep Disorders and Their Impact on Academic Performance Among Saudi University Students: A Cross-Sectional Study

**DOI:** 10.7759/cureus.61334

**Published:** 2024-05-29

**Authors:** Ridwan M Alomri, Yaser Alghamdi

**Affiliations:** 1 Department of Psychology, College of Social Sciences and Media, University of Jeddah, Jeddah, SAU; 2 Department of Educational Psychology, College of Education, Taibah University, Madinah, SAU

**Keywords:** academic, university students, saudi, sleep disorders, prevalence

## Abstract

Background: University students encounter a variety of sleep problems that have an impact on their health and academic performance. Therefore, the aim of this study was to evaluate the prevalence of sleep disorders and their impact on academic performance among Saudi university students.

Methods: This is an online cross-sectional study that involved university students and was conducted between November 2022 and February 2023 in Saudi Arabia. Sleep disorders were examined among undergraduate students through online screening surveys including the Epworth Sleepiness Scale (ESS) to assess excessive daytime sleepiness, the Insomnia Severity Index (ISI) to measure insomnia, and the Berlin questionnaire to examine obstructive sleep apnea (OSA). Restless leg syndrome (RLS) was measured using the RLS rating. SPSS version 29.0 (IBM Corp., Armonk, NY, USA) was used for all statistical analyses. Binary logistic regression analysis was used to identify predictors of having excessive daytime sleepiness, insomnia, sleep-disordered breathing, and RLS.

Results: The sample included 449 participants. According to the ESS, 56.6% (n=254) of students reported excessive daytime sleepiness. Using the ISI, 78.4% (n=352) of students were found to have insomnia. The Berlin questionnaire indicated that 6.7% (n=30) of students had sleep-disordered breathing. Additionally, 13.6% (n=61) of students reported having RLS. Male students had higher odds of sleep-disordered breathing compared to females (p<0.01), while marital status showed that married students had higher odds of sleep-disordered breathing (p<0.05). Students who reported nighttime sleeping had lower odds of insomnia and restless leg syndrome (p<0.05). Having regular sleeping times was associated with higher odds of insomnia (p<0.05). Napping sometimes was associated with higher odds of excessive daytime sleepiness and sleep-disordered breathing (p<0.05). Students working day and night shifts had higher odds of sleep-disordered breathing (p<0.01). Interestingly, students with comorbidities had lower odds of RLS (p<0.01).

Conclusion: This study established that sleep disturbances among students significantly reduce grade point averages (GPAs), where the most affected were male students and those who were married. The government should implement sleep education programs, provide resources for the management of sleep, encourage consistent sleep schedules, and come up with targeted interventions for at-risk groups. More research is also recommended on effective sleep interventions.

## Introduction

University students experience a range of sleep disorders that can affect their well-being and academic achievements [1]. The prevalence of sleep disorders among undergraduate students tends to worsen as time goes on, which is problematic because even the temporary existence of significant sleep disorders is widely recognized to have negative effects on health outcomes [2]. The literature commonly identifies several sleep disorders, including insomnia, hypersomnia, obstructive sleep apnea (OSA), circadian rhythm disturbance, and sleep movement disorder [3].

Academic years can be exceedingly demanding for students. A significant number of students struggle with effectively managing their academic obligations and coordinating their study-life balance, while also expressing apprehension about securing a profession in a highly competitive job market [4]. Consequently, this particular demographic is vulnerable to developing mental health problems during their time in university [5]. These concerns have created a global interest in investigating these challenges by health professionals, academic specialists, and researchers, with the aim of mitigating the effects of these pressures on students' welfare [6]. Furthermore, recent years have seen a decline in the quality of sleep, especially among those in this age range, due to changes in behavior and society [7]. University students experience inconsistent sleep habits as a result of the academic responsibilities and deadlines they face. Sufficient sleep is crucial for human existence and plays a significant influence on both mental and physical health [8]. Moreover, sleep has a crucial role in maintaining optimal cognitive functioning, particularly in regard to attention, perception, and working memory. Working memory is a type of memory that maintains knowledge that is important for the current task while the brain performs other mental processes. It is a crucial cognitive function in the process of learning [4].

A wide range of studies have been carried out globally to examine the correlation between academic achievement and sleep. The research findings consistently demonstrate a positive correlation between improved sleep quality and enhanced academic performance [9]. However, alternative research suggests that this correlation could be adverse [10]. Out of a group of 2,095 people in Saudi Arabia, 33.8% stated that they sleep for less than seven hours every night. Additionally, females were more likely than males to sleep for less than seven hours [11]. Prior research conducted in Saudi Arabia examined the correlation between sleep quality, academic achievement, and stress levels among medical students. However, the findings regarding the impact of sleep on academic performance were inconsistent, despite a high prevalence of poor sleep quality reported at 77%. However, a significant number of Saudi medical students hold the belief that they experience a high standard of sleep [12,13]. Furthermore, some local sources have reported that medical students experience high levels of stress [14]. Based on a comprehensive analysis of existing research, no investigations have been carried out in Saudi Arabia to investigate the prevalence and factors influencing sleep disorders, as well as their connection to academic performance, among university students who are not studying medical subjects. Therefore, the aim of this study was to evaluate the prevalence of sleep disorders and their impact on academic performance among Saudi university students.

## Materials and methods

Study design

This is an online cross-sectional study that involved university students and was conducted between November 2022 and February 2023 in Saudi Arabia.

Study population

Undergraduate students who are currently studying at universities in Saudi Arabia formed the study population. The inclusion criteria for this study were to be an undergraduate student from any level of study and any field of study (whether medical or non-medical field), aged between 18 and 30 years, and to be a Saudi student. There was no restriction based on students' gender. The exclusion criterion was having a history of administration of sleep medications.

Participants' recruitment

The convenience sampling technique was used to recruit the study sample. University students were recruited by sending an online survey to them via social media platforms (Facebook, LinkedIn, and WhatsApp). A request to participate and a link to the survey were administered to the study participants. An informed consent form was provided to participants along with the online survey. The students were requested to provide electronic informed consent before participating in the survey.

Questionnaire tool

Participants were asked to answer sociodemographic questions about their age, gender, marital status, and academic year. The cumulative grade point average (GPA) that each student currently has in their academic record was requested in order to analyze their academic performance. Besides, the study participants were requested to complete the Epworth Sleepiness Scale (ESS) [15], the Insomnia Severity Index (ISI) [16], and the Berlin questionnaire to examine their sleep problems [17]. In addition, the study participants were asked whether they have restless leg syndrome (RLS). Besides, the questionnaire tool examined participants' sleep chorotype and type of shiftwork.

The ESS is an eight-item survey that asks participants to rate how likely it is that they would nod off if they were in various situations that are differentially soporific [15]. These situations include those in which the majority would be expected to nod off and others in which only the most sleep-deprived people would be expected to doze (e.g., while sitting and talking with someone). On a scale ranging from zero (would never nod off) to three (high likelihood of nodding off), participants gave each item a rating. High reliability is present for this measure (α=0.70 in the present sample) [17]. The responses to the eight questions on the ESS were added to represent the individuals' degree of sleepiness; larger numbers represent more sleepiness. A score of 0-7 indicated that it is unlikely that you are abnormally sleepy. A score of 8-9 indicated an average amount of daytime sleepiness. A score of 10-15 indicated that the individual may be excessively sleepy depending on the situation. In this case, the individual may want to consider seeking medical attention. A score of 16-24 indicated that the individual is excessively sleepy and should consider seeking medical attention.

The Insomnia Severity Index is a seven-item self-report questionnaire that evaluates the type, severity, and effects of insomnia [16]. The dimensions assessed include the severity of sleep onset, maintenance, and early morning awakening problems, sleep dissatisfaction, influence of sleep difficulties with daily activities, noticeability of the sleep disorders by others, and distress brought on by the sleep difficulties. Each question is rated on a 5-point Likert scale (0 = no difficulty, 4 = extremely severe problem), resulting in a total score that ranges from 0 to 28. There is no insomnia (0-7), subthreshold insomnia (8-14), moderate insomnia (15-21), and severe insomnia (22-28) according to the total score. The reliability of this questionnaire in the current study sample was high (α=0.69).

Students who have OSA have been identified using the Berlin questionnaire [18]. Ten questions in three categories make up the self-administered survey. High risk in category one was characterized as persistent snoring symptoms in two or more snoring-related questions. For group two, high risk was characterized as ongoing drowsiness during the day, sleepy driving, or both. A history of hypertension or a body mass index (BMI) of more than 30 kg/m^2^ were both considered indicators of high risk in category three. The OSA high-risk participants were those who met the criteria for high risk in at least two out of three categories. The reliability of this questionnaire in the current study sample was good (α=0.59).

Ethical approval

This study was approved by the Bioethics Committee of Scientific and Medical Research at the University of Jeddah, Jeddah, Saudi Arabia (UK-REC-177). All participants provided their written consent before participating in the study.

Statistical analysis

SPSS version 29.0 (IBM Corp., Armonk, NY, USA) was used for all statistical analyses. For continuous data, means (standard deviation (SD)) were used. Frequencies and percentages were used to present categorical variables. According to their GPA, the students were divided into three groups (out of five): lower than 3.0, 3.0-4.0, and >0.4. Binary logistic regression analysis was used to identify predictors of having excessive daytime sleepiness, insomnia, sleep-disordered breathing, and RLS. Odds ratios (ORs) with a 95% confidence interval were used to present the findings of the regression analysis. The results were deemed statistically significant at p<0.05 in all two-sided statistical analyses.

## Results

Characteristics of the study participants

Table 1 presents the sociodemographic characteristics and sleeping habits of the study participants. The sample included 449 participants, with 61.7% (n=277) female. The participants had a mean age of 21 years, with a standard deviation of 2.3 years. The majority of participants were single (93.1%; n=418). Regarding the academic year, the highest proportion was in the first year (23.6%; n=106). In terms of sleeping habits, most participants reported no specific time for sleeping (45.2%; n=203), followed by nighttime sleeping (50.1%; n=225). Additionally, a minority reported using sleeping pills (12.9%; n=58), having RLS (13.6%; n=61), or having regular sleeping times (18.9%; n=85). The results regarding napping habits show that the majority of participants (56.6%; n=254) reported taking a nap sometimes. In terms of shift work, the majority (90.2%; n=405) reported not working in shifts. Additionally, 14% (n=63) of participants reported having comorbidities.

**Table 1:** Sociodemographic characteristics of the study participants SD: standard deviation

Sociodemographic characteristics	Number	%
Gender		
Male	172	38.3%
Female	277	61.7%
Age (years)		
Mean±SD	21.0±2.3	
Marital status		
Single	418	93.1%
Married	26	5.8%
Separated	5	1.1%
Academic year		
First year	106	23.6%
Second year	83	18.5%
Third year	97	21.6%
Fourth year	97	21.6%
Fifth year or higher	66	14.7%
Usual sleeping time		
Daytime	21	4.7%
Nighttime	225	50.1%
No specific time	203	45.2%
Do you use sleeping pills?		
Yes	58	12.9%
Do you have restless leg syndrome?		
Yes	61	13.6%
Do you have regular sleeping time?		
Yes	85	18.9%
Do you take a nap usually?		
Yes, sometimes	254	56.6%
Yes, daily	58	12.9%
No	137	30.5%
Do you work in shifts?		
Yes (day and night shifts)	34	7.6%
Yes (night shift)	10	2.2%
No	405	90.2%
Do you have comorbidities?		
Yes	63	14%

Prevalence of sleep disorders among Saudi college students

Table 2 presents the prevalence of different sleep disorders among Saudi university students using various evaluation scales. According to the ESS, 56.6% (n=254) of students reported excessive daytime sleepiness. A total of 73 (16.3%) students were estimated to have an average amount of daytime sleepiness, 202 (45%) students were estimated to have excessive daytime sleepiness, and 52 (11.6%) students were estimated to have severe excessive daytime sleepiness. Using the ISI, 78.4% (n=352) of students were found to have insomnia. A total of 183 (40.8%) students were estimated to have subthreshold insomnia, 133 (29.6%) students were estimated to have clinical insomnia (moderate severity), and 36 (8%) students were estimated to have clinical insomnia (severe). The Berlin questionnaire indicated that 6.7% (n=30) of students had sleep-disordered breathing. Additionally, 13.6% (n=61) of students reported having RLS.

**Table 2:** Prevalence of different sleep disorders among Saudi university students

Sleep disorders evaluation scale	Number	%
Epworth Sleepiness Scale		
Excessive daytime sleepiness	254	56.6%
Insomnia Severity Index		
Insomnia	352	78.4%
Berlin questionnaire		
Sleep-disordered breathing	30	6.7%
Restless leg syndrome		
Yes	61	13.6%

Sleep disorders and academic performance

Table 3 illustrates the difference between different sleep disorders, assessed by various scales, and academic performance among university students, as indicated by their GPAs. For the ESS, a significant association was found between excessive daytime sleepiness and GPA, with 65.5% (n=137) of students with a GPA of >4 reporting daytime sleepiness compared to 28.7% (n=60) with a GPA of >3 to 4 and 5.7% (n=12) with a GPA of <3 (p=0.05). Similarly, the ISI showed a strong association with GPA, with 60.7% (n=207) of students with a GPA of >4 reporting insomnia compared to 30.5% (n=104) with a GPA of >3 to 4 and 8.8% (n=30) with a GPA of <3 (p<0.001). In contrast, the Berlin questionnaire did not show a significant association with GPA for sleep-disordered breathing (p=0.82). However, RLS was significantly associated with GPA, with 44.3% (n=27) of students with a GPA of >4 reporting RLS compared to 45.9% (n=28) with a GPA of >3 to 4 and 9.8% (n=6) with a GPA of <3 (p=0.01).

**Table 3:** Sleep disorders and academic performance GPA: grade point average

Scale	GPA>4	GPA>3 to 4	GPA<3	p-value
Epworth Sleepiness Scale				
Excessive daytime sleepiness	137 (65.5%)	60 (28.7%)	12 (5.7%)	0.05
Insomnia Severity Index				
Insomnia	207 (60.7%)	104 (30.5%)	30 (8.8%)	<0.001
Berlin questionnaire				
Sleep-disordered breathing	19 (63.3%)	8 (26.7%)	3 (10%)	0.82
Restless leg syndrome				
Yes	27 (44.3%)	28 (45.9%)	6 (9.8%)	0.01

Predictors of sleep disorders among university students

Table 4 presents the odds ratios of sociodemographic characteristics and habits as predictors of different sleep disorders among university students. The results suggest that various factors, including gender, marital status, sleeping habits, and shift work, may influence the likelihood of experiencing different sleep disorders among university students. Male students had higher odds of sleep-disordered breathing compared to females (p<0.01), while marital status showed that married students had higher odds of sleep-disordered breathing (p<0.05). Students who reported nighttime sleeping had lower odds of insomnia and restless leg syndrome (p<0.05). Having regular sleeping times was associated with higher odds of insomnia (p<0.05). Napping sometimes was associated with higher odds of excessive daytime sleepiness and sleep-disordered breathing (p<0.05). Students working day and night shifts had higher odds of sleep-disordered breathing (p<0.01). Interestingly, students with comorbidities had lower odds of RLS (p<0.01).

**Table 4:** Predictors of sleep disorders among university students *p<0.05, **p<0.01, ***p<0.001

Sociodemographic characteristics	Odds ratio of having excessive daytime sleepiness	Odds ratio of having insomnia		Odds ratio of having sleep-disordered breathing	Odds ratio of having restless leg syndrome
Gender					
Female	1.00				
Male	0.96 (0.66-1.41)	1.51 (0.93-2.44)		3.36 (1.58-7.17)**	0.64 (0.35-1.14)
Age category					
Younger than 21 years	1.00				
21 years and older	1.03 (0.71-1.50)		0.70 (0.44-1.11)	1.15 (0.56-2.40)	0.91 (0.53-1.56)
Marital status					
Single	1.00				
Married	1.28 (0.58-2.84)	0.59 (0.25-1.41)		3.45 (1.21-9.86)*	1.54 (0.56-4.25)
Separated	0.28 (0.03-2.48)	0.39 (0.07-2.40)		-	-
Academic year					
First year	1.00				
Second year	0.62 (0.35-1.11)	0.78 (0.39-1.59)		0.62 (0.20-1.88)	1.58 (0.71-3.53)
Third year	0.73 (0.42-1.27)	1.09 (0.54-2.24)		0.41 (0.13-1.36)	1.11 (0.49-2.52)
Fourth year	1.30 (0.75-2.27)	0.60 (0.31-1.17)		0.98 (0.38-2.53)	1.11 (0.49-2.52)
Fifth year or higher	1.33 (0.72-2.46)	0.86 (0.40-1.86)		0.62 (0.19-2.06)	0.85 (0.32-2.25)
Usual sleeping time					
Daytime	1.00				
Nighttime	1.45 (0.58-3.63)	0.11 (0.01-0.82)*		0.49 (0.13-1.83)	0.28 (0.09-0.85)*
No specific time	1.58 (0.63-3.97)	0.36 (0.05-2.77)		0.38 (0.10-1.46)	0.74 (0.25-2.14)
Do you have regular sleeping time?					
No	1.00				
Yes	1.56 (0.96-2.52)	8.34 (4.9-14.1)***		1.28 (0.48-3.43)	1.64 (0.75-3.59)
Do you take a nap usually?					
No	1.00				
Yes, daily	0.66 (0.43-1.02)	1.46 (0.83-2.56)		1.62 (0.71-3.73)	1.67 (0.94-2.97)
Yes, sometimes	1.83 (1.01-3.30)	2.06 (0.87-4.90)		2.95 (1.16-7.49)*	0.86 (0.34-2.18)
Do you work in shifts?					
No	1.00				
Yes (night shift)	1.10 (0.31-3.85)	0.68 (0.17-2.67)		4.15 (0.83-20.68)	1.78 (0.37-8.60)
Yes (day and night shifts)	0.98 (0.48-1.97)	3.00 (0.90-10.02)		4.31 (1.70-10.93)**	2.56 (1.13-5.79)*
Do you have comorbidities?					
No	1.00				
Yes	1.16 (0.68-1.99)	0.56 (0.27-1.19)		0.55 (0.23-1.34)	0.39 (0.20-0.74)**

Association between sleep disorders and academic achievement

Binary logistic regression analysis identified that there is no significant association between having excessive daytime sleepiness (OR: 1.63 (95% confidence interval: 0.87-3.06); p=0.128), insomnia (OR: 0.41 (95% confidence interval: 0.16-1.07); p=0.070), sleep-disordered breathing (OR: 1.70 (95% confidence interval: 0.62-4.65); p=0.303), or RLS (OR: 0.95 (95% confidence interval: 0.38-2.34); p=0.910) and academic achievement.

## Discussion

In our study, we found that 56.6% (n=254) of students reported excessive daytime sleepiness. Using the ISI, 78.4% (n=352) of students were found to have insomnia. The Berlin questionnaire indicated that 6.7% (n=30) of students had sleep-disordered breathing. Additionally, 13.6% (n=61) of students reported having RLS. These results show the heightened prevalence of sleep disorders among university students in Saudi Arabia, making academic settings the first line to boost awareness of the health of sleep. Aligning with our findings, earlier studies have noted elevated rates of excessive daytime sleepiness among college students, ranging from 25% to 47.8% [13,19-22]. Prior investigations have reported factors including lifestyle habits, area of residence, socioeconomic status, family type, academic year, and age that were impacting incidences of excessive daytime sleepiness among the student population [23,24].

Regarding insomnia prevalence, our findings aligned with the existing literature, reporting rates ranging from less than 20% in some countries to 70% in others [13,25-33], with considerable variation depending on methodological and demographic factors.

The prevalence of sleep-disordered breathing in our study was significantly lower than among "medical students, interns, and residents" in Bangkok, where the overall prevalence of increased risk of sleep-disordered breathing was much higher, reaching 36.5% [34]. This considerable difference underscores the variation in the prevalence of sleep-disordered breathing between these two groups of students in different geographic locations. Besides, disturbed sleep-disordered breathing depends on risk factors such as snoring, body mass index (BMI), age, and gender [35].

Our findings about the prevalence of RLS among university students in Saudi Arabia (13.6%; n=61) are consistent with earlier investigations that document that RLS prevalence among university students ranges from 8% to 23% [36,37]. These findings underscore the high burden of sleep disturbances among university students in Saudi Arabia and global populations. It also highlights the necessity for targeted interventions to help lessen the problem and enable healthier lifestyles among this population.

Our study has shown a significant association between students' sleep disturbance and academic performance. Particularly, we found a significant association between excessive daytime sleepiness and GPA, with 65.5% (n=137) of students with a GPA of >4 reporting daytime sleepiness compared to 28.7% (n=60) with a GPA of >3 to 4 and 5.7% (n=12) with a GPA of <3 (p=0.05). Besides, the ISI showed a strong association with GPA, with 60.7% (n=207) of students with a GPA of >4 reporting insomnia compared to 30.5% (n=104) with a GPA of >3 to 4 and 8.8% (n=30) with a GPA of <3 (p<0.001). These aligned with the previous studies that emphasize the critical impact of excessive daytime sleepiness on many aspects of daily functioning, such as academic and cognitive performance [19,38]. Excessive daytime sleepiness is considered a central public health problem that may interfere with a person's ability to engage in daily activities and can cause life-threatening accidents [19,38]. It is associated with many mental and public health problems and is influenced by factors that affect sleep quality [39,40]. Studies have established a strong association between excessive daytime sleepiness and poor academic performance among university students [41]. Nevertheless, findings concerning the association between excessive daytime sleepiness and academic performance vary across diverse populations. While numerous studies found that excessive daytime sleepiness had a strong association with poor academic performance [41], others have found no significant association between the two [42].

Research consistently finds that sleep disorders are associated with an increased risk of poor academic performance and usually lower mean GPA (<2.0) [19]. On the contrary, regular sleeping students tend to have high GPAs, which shows that adequate sleep is relevant to students' academic success [43]. In line with our findings, insomnia is a common sleep disorder among university students in the United States and Saudi Arabia, and it leads to poor academic grades [19,44]. Moreover, insomnia levels vary significantly with a range of demographic characteristics, such as GPA, marital status, smoking status, year of study, and gender [45]. The influence of sleep disorders (excessive daytime sleepiness and insomnia) on academic performance among college students highlights the requirement for intervention to enhance academic performance and mental and general well-being.

In line with our findings, among medical students, sleep apnea was not associated with GPA in Iraq [45]. Among male university students, obstructive sleep apnea was not associated significantly with GPA in Oman [45]. On the other hand, obstructive sleep apnea was not associated significantly with lower GPA among female university students in Oman [45]. Sleep-related breathing is strongly connected with lower academic performance among schoolchildren [46]. Several factors, including age, gender, socioeconomic status, race, and BMI, can impact the association between sleep-disordered breathing [35,47,48] and academic performance, which needs additional investigation.

The present investigation found that RLS was significantly associated with GPA, with 44.3% (n=27) of students with a GPA of >4 reporting RLS compared to 45.9% (n=28) with a GPA of >3 to 4 and 9.8% (n=6) with a GPA of <3 (p=0.01), indicating that students with RLS may have difficulty achieving higher grades. These are consistent with prior literature suggesting considerable adverse effects of RLS on sleep and cognitive performance [49,50]. Previous studies show that RLS impairs sensitive cognitive functions in response to sleep deprivation, raised daytime fatigue, and decreased sleep efficiency and total sleep time [51]. Moreover, the results of prior studies also indicate that individuals with RLS appear to have cognitive disorders relative to individuals who do not have the disorder. An earlier investigation reported that high and middle school students who experience pain or restlessness in their legs while sleeping have poor academic performance (lower GPA). Studies involving Turkish medical students revealed a significant relationship between RLS and low GPA [49,51], especially with excessive daytime sleepiness.

In our study, we identified significant associations between sleep-disordered breathing and gender and marital state. Male students had higher odds of sleep-disordered breathing than females (p<0.01), and those who were married were at higher odds of sleep-disordered breathing than the unmarried students (p<0.05). The gender differences in the prevalence of sleep-disordered breathing are consistent with prior studies, which show a higher sleep-disordered breathing prevalence among males than females in Wisconsin, Japan, and America [48]. The variables that could contribute to this gender disparity involve disparities in hormones, fat distribution, arousal response, neurochemical mechanisms, and anatomy of the upper airway [52]. In line with our findings regarding associations between sleep-disordered breathing and marital state, a prior study conducted in Canada found that marital state (married) had significant associations with sleep apnea [53]. However, an earlier study revealed that the chances of survival for patients diagnosed with obstructive sleep apnea are better for married patients [54]. Accordingly, it is essential to reduce the burden associated with respiratory-related sleep disturbances and develop targeted interventions and prevention strategies based on demographic predictors of sleep-disordered breathing among university students. Targeted screening programs for at-risk populations, such as male students and married students, may help in the early detection and treatment of sleep disorders.

The findings of our study indicate lower odds of insomnia and RLS among students who reported night-time sleeping (p<0.05). These findings are consistent with earlier studies, which indicate that the circadian clock has an essential function in sleep and wake regulation. The principal function of the circadian clock is to encourage wakefulness during the day and enable nighttime consolidation of sleep [55]. Chronic insomnia has been linked with abnormality in the endogenous circadian clock, which might be running at a slower or faster rate as compared to regular [56]. Besides, many studies attributed RLS to genetic factors, mainly concerning the *CLOCK* gene, which controls all circadian rhythms [57]. Individuals who delay their bedtimes report an increased risk for insomnia, poorer quality of sleep, and higher levels of symptoms of anxiety and depression [58]. This evidence highlights the complex relations of circadian rhythms, genetic factors, and disturbances in sleep, with underlying importance in night-time sleep for health and well-being.

Contrary to expectations, we found that having regular sleeping times was associated with higher odds of insomnia among Saudi university students (p<0.05). These suggest that a rigid consistency in fixed sleep schedules may not necessarily translate into better sleep quality or fewer sleep disturbances. Environmental factors, culture, physiological and psychological conditions, and age affect sleep quality. In addition, factors that may significantly reduce sleep quality include sweating during sleep, rapid temperature changes, and skin temperature [59], thus indicating the importance of understanding individual variability in sleep behaviors and outcomes.

Many factors may explain the association between regular sleep times and a higher incidence of insomnia. Students who are very strict with fixed sleep schedules may be exposed to increased stress or pressure to sleep on time, which may, in turn, increase sleep-related arousal and increase problems in the onset and maintenance of sleep. In line with these, a previous study indicated that poor sleep quality has a significant link with stress [12]. Evidence has also highlighted that microstructural and macrostructural changes in sleep are closely related to cognitive and physiological arousal, respectively [60]. Much previous research has indicated that emotional, cognitive, and physiological hyperarousal results in chronic insomnia because it interferes with sleep among some individuals [61].

Our results indicated that napping sometimes was associated with higher odds of excessive daytime sleepiness and sleep-disordered breathing (p<0.05). These are consistent with previous research suggesting that among young adults, napping may be detrimental to the overall quality of sleep besides increased alertness during the afternoon [62]. Research has also noticed that frequent napping is associated with other disorders, including circadian rhythm- and sleep-related disorders, and regular napping increases mortality risk [63]. Additionally, napping is related to sleep-disordered breathing as a diagnostic marker for conditions such as sleep apnea [64]. Excessive daytime sleepiness is the most prevalent cause of napping among Australian university students [65]. In addition, among Spanish university students, excessive daytime sleepiness was among the substantial napping-related consequences variables [66]. Thus, our findings add to a deeper understanding of the complex association between daytime napping and sleep health.

Our study demonstrated higher odds of sleep-disordered breathing among students working day and night shifts (p<0.01). Our findings highlight the intricate impact of lifestyle and health conditions on the occurrence of sleep disorders among university students in Saudi Arabia. Besides, shift work/sleep-disordered breathing associations highlight the adverse effects of shift work on sleep quality and respiratory health among university students. In line with our findings, prior research underlines that shift workers often suffer from sleep disorders including sleep apnea [67]. Furthermore, shift work that incorporates rotating may significantly affect normal circadian rhythms [68], indicating the existence of an imbalance in the body's internal clock [69], consequently affecting all physiological processes regulated by the body's circadian rhythms, including sleeping. Thus, it impairs working performance and also poses risks to mental health, leading to more severe health outcomes [70].

Regarding our findings that students with comorbidities had lower odds of RLS (p<0.01), this unexpected result indicates that comorbidities may act as protective factors against RLS. The presence of comorbidities might modify the neurochemical or physiological mechanisms contributing to the pathophysiology of RLS, resulting in a lower likelihood of RLS symptoms [71-74]. Likewise, comorbid individuals could seek medical consultation and care more than non-comorbid ones for their overall health problems, which might indirectly reduce symptoms of RLS through better-controlling health conditions and related risk factors. For example, among those with cardiovascular diseases (CVDs), following a healthy lifestyle can manage CVD and reduce the risk of developing CVD [75], and this may also lower the risk of RLS symptoms. Indeed, a prior study confirmed these, establishing a link between a healthy lifestyle and a lower likelihood of developing RLS [76]; this is related to factors such as preserving a healthy weight, regular physical activity, and stopping smoking [76].

This study has limitations. The cross-sectional study design restricted the ability to examine causality across the study variables. The use of an online survey might have affected the generalizability of our study findings as we might have missed some targeted study populations who do not have access to online platforms. However, we believe that the possibility of this limitation is minimal as the targeted study population "university students" are one of the major users of social media platforms all over the world.

## Conclusions

Our study highlights a high prevalence of sleep disturbances among students, with a significant impact on academic performance. Excessive daytime sleepiness, insomnia, sleep-disordered breathing, and restless leg syndrome were common among the student population. Notably, daytime sleepiness and insomnia were strongly associated with lower GPAs, indicating the potential negative impact of sleep disturbances on academic achievement. Male students and those who were married were more likely to experience sleep-disordered breathing, emphasizing the need for targeted interventions in these groups. Future research should focus on exploring effective interventions to improve sleep quality among students and evaluating their impact on academic performance.
